# Indigenous Knowledge of Herbal Medicines among Adolescents in Amassoma, Bayelsa State, Nigeria

**DOI:** 10.5539/gjhs.v8n1p217

**Published:** 2015-05-20

**Authors:** Gideon O. Alade, Ese Okpako, Kola’ K. Ajibesin, Olanrewaju R. Omobuwajo

**Affiliations:** 1 Department of Pharmacognosy & Herbal Medicine, Faculty of Pharmacy, Niger Delta University, Wilberforce Island, Bayelsa State, Nigeria

**Keywords:** herbal medicines, indigenous knowledge, secondary schools, Niger Delta

## Abstract

**Background:**

The use of herbal medicines in Nigeria is on the increase. Documented Population based data on the use of herbal medicinal products and indigenous knowledge among the younger generations are lacking in Nigeria and Africa at large.

**Aim:**

The aim of this study is to investigate the extent of use and general knowledge of herbal medicines among adolescents in the Niger Delta Region of Nigeria.

**Methods:**

The study covered a total of Two hundred and twenty-eight adolescents randomly selected in Senior Secondary Schools (SSS 1-3) in Amassoma using a semi structured questionnaire/Interview and informal conversation on the respondents.

**Findings:**

Nearly all (97%) the respondents have had contact with herbs. Less than 1% had contact with herbs through formal education (teachers/literatures). Stimulation of interest was majorly through parents (53%). Grandparents were the highest (46%) of custodian of indigenous knowledge. Parents were the next (39.7%). Only 39% of the respondents would prefer the use of herbal medicine to modern medicine. Fever was the main ailment mentioned followed by eye ailment and stomach ache. *Vernonia amygdalina* was the main plant for the treatment of fever.

**Conclusion:**

The study revealed that parents are the major custodians of knowledge being transferred to the younger generation and little or none is learnt from Schools. There is therefore the need to include the study of herbal medicines in School’s curricula especially at SS 2 and SS 3 since they are matured enough to appreciate the importance of Herbal medicine so as to prepare them for the promotion of herbal medicine in future and to preserve our indigenous knowledge.

## 1. Introduction

Herbal medicines are drugs made from herbs or plants. They are also commonly referred to as phytomedicines, plant medicines, green medicines, traditional medicine potions, traditional remedies, plant drugs and forest health products among others (Osemene, 2011; Elujoba, 1998). They are also referred to as finished labelled medicinal products that contain as active ingredients aerial or underground parts of plants or other plant materials or combinations thereof whether in the crude state or as plant preparations (WHO, 1996). Plant products include juices, gums, fatty oils and other secondary metabolites such as alkaloids, flavonoids, anthraquinones, saponins among others. They may also contain standard excipients in addition to the active ingredients. Exceptionally, in some countries herbal medicine may also contain by tradition, natural organic or inorganic active ingredients which are not of plant origin (WHO, 1996). Over 80% of people living in developing countries depend on herbal medicines as their immediate choice in the treatment of diseases showing its relevance and importance in Primary Health Care (Moody, 2007). In 1976, about a quarter of the prescription drugs dispensed by community pharmacy in the United States contained at least one active ingredient derived from plants (Farnsworth & Morries, 1976). Currently, dispensing herbs/active ingredients is on the increase as herbal medicine is becoming more popular (Ekor, 2013). The WHO estimate of population that has used some form of alternative or complementary medicine including Ayurvedic, homeopathic, naturopathic, traditional oriental and Native American Indian medicine in developing countries is between 70 and 80 % (Oreagba & Oshikoya, 2011).

Herbal medicines have been recognised by the WHO as the most popular form of traditional medicine, and thus, highly lucrative in the international medicine market. Annual revenues in Western Europe were estimated at US# 5 billion in 2003-2004, in China the revenue was estimated at US# 14 billion in 2005, and in Brazil it was US# 160 million in 2007 (Oreagba & Oshikoya, 2011). The increasing widespread use of Traditional medicine has prompted the WHO to promote its integration into the national health care systems of some countries and to encourage the development of national policy and regulations as essential indicators of the level of integration of such medicine into a national health care system (Oreagba & Oshikoya, 2011). Also in Nigeria, approximately 205 medicinal plant species are prevalent in nature (FEPA, 1992). Traditional medicine in Nigeria is as old as the people and it is growing in importance and this has made the Federal Government of Nigeria to formulate a traditional medicine policy and to establish a Traditional Medicine Council to regulate practice and encourage research in five core areas (herbal medicine, bone setting, mental health, traditional birth attendance and sale of traditional medicine ingredients (Chesa, 2006). Herbal medicines are by far less concentrated, less toxic and are used in much lower doses than orthodox medicine which in its concentrated drug formulations are designed to target and reverse specific pathologies in the minimum of time (Osemene, 2011; Ohuabunwa, 1998; Moody, 2007). The plants used in herbal medicine have been found to carry their own in-built safety mechanisms. Furthermore, they are ideal tools to restore damaged physiological processes since they consist of a multiplicity of chemical components which act synergistically to make active constituents bio available or to buffer the otherwise potentially powerful active principles thus preventing harmful side effects (Osemene, 2011, Moody, 2007). Herbal medicine has its root in prehistory making every bit as ancient tradition as farming or cooking. In the Graeco-Roman era, Hippocrates (father of medicine), Theophrastus (father of Botany), Galen (originator of pharmaceutical galenicals) and Dioscoroides were all herbalists (Osemene, 2011; Moody, 2007). Globally, people developed unique indigenous healing traditions adapted and defined by their culture, beliefs and environment, which satisfied the health needs of their communities over centuries (Oreagba & Oshikoya, 2011). Previous studies of herbal medicine use in Nigeria were focused on adults with various forms of chronic illnesses (Danesi & Adetunji, 1994; Amira & Okubadejo, 2007; Ogbera et al., 2010), pregnant women (Fakeye et al., 2009) and children with chronic illnesses (Oshikoya et al., 2008) and among a general population without chronic health conditions. No study has ever been evaluated in Nigeria or other African countries on younger population to know if there is transfer of knowledge of herbal medicine from the older population to the younger ones of age range between 14 and 18 years. This study was therefore aimed to assess the extent of use and the general knowledge, benefits and safety of herbal medicines among Senior Secondary School Students resident in Amassoma in the Niger Delta region of Nigeria.

## 2. Methodology

**Description of Study Area**

**Figure 1:**
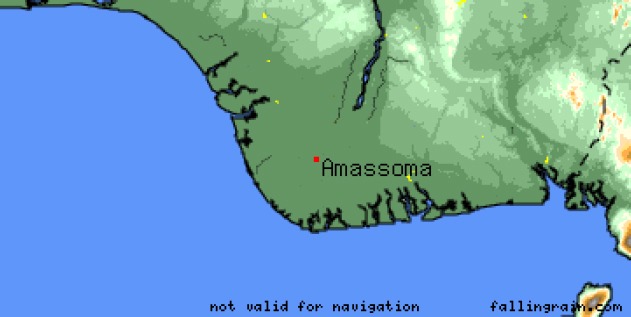
Map of Southern Ijaw showing Amassoma

Amassoma is the head quarter of Ogboin clan as well as Ogboin-North Rural Development Authority in the Southern Ijaw Local Government Area of Bayelsa State (Figure 1). It is the host community to the Niger Delta University, Wilberforce Island Bayelsa. It is located about 40 km to the South of Yenagoa; the State capital. It is on an altitude of 512 above sea level, bounded in the North by River Nun, West by Otuan, East by Toru Ebeni and the South by Ogobiri. It is the biggest town in Southern Ijaw Local Government Area. The area has a coastline of approximately 60 km on the Bight of Bonny. It has an area of 2,682 km^2^ and a population of 319,413 (Federal Government of Nigeria, 2007)

The study covered the only two Secondary Schools having Senior (SS 1 to SS 3) classes in Amassoma. Students in the Senior Secondary classes were involved. Permission was sought from the School management. A total of Two hundred and twenty-eight (228) students were present in these classes. Only 164 (72%) students responded and finally selected for the study through a purposive and convenience sampling process. Furthermore, semi structured questionnaires/interviews amidst informal conversation on the respondents whose opinions were sought on their knowledge of herbal medicine. The questionnaires had two components: Demography and Indigenous knowledge on herbal medicines. The participants were allowed to give the vernacular names of the plants while some were identified at site. The results of the pre-tested questionnaires were used to make necessary modifications and corrections on the questionnaires and interview guides. Data were analysed using descriptive statistics such as frequency and percentages.

## 3. Results and Discussion

**Demography**

Males were more in all the three arms of the classes with 54% except in S. S. S 2 with a higher number of females (54%). It has been reported that Bayelsa state is among the States in Nigeria with low girl child education probably due to under age child bearing and poverty (Punch Newspaper, Oct., 2013). Age range of 14 – 18 years was the highest in terms of the number of respondents (86% of students in this category) and the majority (82%) speaks Ijaw language apart from English which is the medium of communication in Nigerian schools (Table 1).

**Table 1:** Demographical Data of the study Population

	SS1	SS2	SS3	TOTAL
GENDER				
MALE	50	19	20	89 (54 %)
FEMALE	35	21	19	75 (46 %)
AGE				
9 TO 13	7	1	1	9 (6 %)
14 TO 18	75	37	30	142 (86 %)
> 19	3	2	8	13 (8 %)
LANGUAGE				
IJAW	69	36	30	135 (82.2 %)
YORUBA	0	0	2	2 (1.2 %)
IBO	5	0	0	5 (3.1 %)
HAUSA	0	0	0	0 (0 %)
OTHERS	2	1	2	5 (3.1 %)
COMBINATION	9	3	5	17 (10.4 %)

### 3.1 Contact With Herbs

Nearly all the respondents (97%) have had contact with herbs. The highest medium of contact was through usage (approximately 40%), 25% acquired knowledge on herbs through their parents. This is different from a report in which 80% acquired their knowledge through parents among Secondary and Grammar School students in Slovenia (Strgar et al., 2013). While 37% had contact through a combination of usage, parents, Teachers and media, only less than 1 % had it through their teachers/literature. This agrees with the case in Slovenia in which it was found that little or nothing on the subject was learnt in school (Strgar et al., 2013). Their stimulation of interest was majorly from their parents (53%), followed by their Grand parents (22%). Others were through combinations of parents, grandparents, herbalists and other relations. This showed that parents are still eager to transfer their indigenous knowledge to their children. Twenty percent of respondents believed that their interest in herbal medicine is high, while majority (52%) showed a middle rating in their interest and 27% showed low interest. Approximately 90% has used herbal medicines at one time or the other whether once, a few times, frequently or occasionally, while only 10% have never taken it before. Out of the 90%, about 26% takes it frequently. Out of these 90% users, approximately 70% are highly satisfied with the effectiveness of herbal medicine. The remaining had a low satisfaction (31%) (Table 2).

**Table 2:** Contact of Respondents to herbal medicines

	SS 1	SS 2	SS 3	TOTAL
CONTACT WITH HERBS				
YES	80	40	39	159 (97 %)
NO	5	0	0	5 (3.1 %)
MEDIUM OF CONTACT				
USAGE	23	13	27	63 (39.6 %)
PARENTS	10	5	25	40 (25.2 %)
TEACHERS/LITERATURE	1	0	0	1 (0.6 %)
MEDIA	11	3	0	14 (8.8 %)
OTHERS	3	1	0	4 (2.5 %)
COMBINATION	27	10	0	37 (23.3 %)
STIMULATION OF INTEREST				
PARENTS	48	19	20	87 (53 %)
GRAND PARENTS	17	9	10	36 (22 %)
RELATIONS	4	3	2	9 (5.5 %)
HERBALIST	0	0	1	1 (0.6 %)
OTHERS	6	6	6	18 (11 %)
COMBINATION	10	3	0	13 (7.9 %)
NONE				
LEVEL OF INTEREST				
LOW	24	11	10	45 (27.4 %)
MIDDLE	41	20	25	86 (52.4 %)
HIGH	20	9	4	33 (20.1 %)
NONE	0	0	0	0 (0 %)
NO OF TIMES HERBS HAVE BEEN TAKEN				
ONCE	22	2	7	31 (19.5 %)
FEW	16	13	22	51 (32.1 %)
FREQUENT	18	18	6	42 (26.4 %)
OCCASSIONAL	19	2	0	21 (13.2 %)
NONE	8	2	4	14 (8.8 %)
LEVEL OF SATISFACTION				
LOW	23	8	14	45 (31 %)
HIGH	50	28	22	100 (69 %)
NONE	0	0	0	0 (0 %)

### 3.2 Knowledge of Medicinal Plants

Grandparents were the highest (46%) custodian of knowledge on medicinal plants in their family followed by their parents with about 40%. Out of the latter, mothers had more knowledge on medicinal plants than fathers with approximately 60%. Women have been recognized as users and custodians of plant biodiversity. In countries like Bolivia, Colombia, Peru, Viet Nam, Indonesia and India, they are responsible for the selection, improvement and storage of seeds and management. Women from sub-Saharan Africa grow over 120 different plant varieties in small areas alongside cash crops (Deda & Rubian, 2004). In a study in Sierra Leone, women could name 31 uses of trees on fallow land and in the forest, while men named eight different uses (Aguilar, 2004). Women have a unique relationship with biodiversity across the globe; they predominate as wild plant gatherers, home gardeners, plant domesticators, herbalists and seed custodians.

Majority (55%) would prefer modern medicine to herbal medicines (39%), while 6% would prefer to opt for both since from their perception, they work synergistically. Out of the 39% that preferred herbal medicine, 61% of them would prefer herbal medicine for its effectiveness, affordability, accessibility and safety. Only 43% would like to attend a course on herbal medicine if there was an opportunity as against about 94% in the case of Slovenia probably because they think herbal medicine is enmeshed in esoterism or does not yet enjoy official recognition by Government. Thirty-one percent (31%) gave a positive response that they would like to practise herbal medicine. A previous report has shown that the younger generation does not seem to have much trust in the traditional medicine system which may be attributed to increasing use of allelopathic medicines which are readily available and considered potent. Up to 85% of respondents had knowledge of medicinal plants and about 95% of these medicinal plants mentioned grow or are cultivated around the home. Approximately 90% could identify the common plants growing around them, 83.5% of them can prepare them for use and up to 90% of these has at one time or the other been involved in self-treatment or prescription (Table 3).

**Table 3:** Knowledge of herbal medicine by the Respondents

	SS 1	SS 2	SS 3	TOTAL
BEST KNOWLEDGE OF MEDICINAL PLANTS IN THE FAMILY				
MOTHER	17	10	11	38 (24 %)
FATHER	11	4	10	25 (15.7 %)
GRAND PARENTS	45	16	12	73 (46 %)
OTHERS	8	6	3	17 (10.7 %)
COMBINATION	1	3	0	4 (2.5 %)
NONE	0	0	3	3 (1.9 %)
PREFERENCE OF MEDICINE				
HERBAL MEDICINE	35	16	13	64 (39 %)
MODERN MEDICINE	46	21	23	90 (55 %)
EQUAL	4	3	2	9 (5.5 %)
NONE	0	0	1	1 (0.6 %)
REASON				
HERBAL MEDICINE				
EFFECTIVE	12	8	7	27 (42.2 %)
AFFORDABLE	2	1	0	3 (4.7 %)
ACCESSIBLE	4	1	1	6 (9.4 %)
NATURAL AND SAFE	5	2	0	7 (4.3 %)
COMBINATION	12	4	5	21 (32.8 %)
MODERN				
EFFECTIVE	27	11	10	48 (53.3 %)
ACCESSIBLE	4	2	1	7 (7.8 %)
SUBJECTED TO SCIENTIFIC RESEARCH	5	2	5	12 (13.3 %)
PRESENTABLE	3	2	1	6 (6.7 %)
CIVILIZATION	1	0	0	1 (1.1 %)
SAFE	0	1	1	2 (2.1 %)
COMBINATION	6	3	5	14 (15.6 %)
EQUAL				
BOTH CAN WORK TOGETHER	4	3	2	9
NONE				
RELIGIOUS BELIEF	0	0	1	1
COURSE ON HERBAL MEDICINE				
YES	35	20	15	70 (42.7 %)
NO	50	20	24	94 (57.3 %)
PRACTICE OF TRADITIONAL MEDICINE				
YES	25	15	10	50 (30.5 %)
NO	60	25	29	114 (69.5 %)
KNOWLEDGE OF MEDICINAL PLANTS				
YES	71	35	33	139 (84.8 %)
NO	14	6	5	25 (15.2 %)
ARE THEY CULTIVATED AROUND THE HOME				
YES	63	35	34	132 (95 %)
NO	6	0	1	7 (5 %)
PLANT IDENTIFICATION				
YES	55	35	34	124 (89.2 %)
NO	12	0	3	15 (10.8 %)
PREPARATION OF HERBAL MEDICINE				
YES	53	31	32	116 (83.5 %)
NO	14	4	5	23 (16.5 %)
SELF TREATMENT/PRESCRIPTION				
YES	48	30	26	104 (90 %)
NO	2	5	5	12 (10 %)

### 3.3 Plant Species

Fever was the main ailment treated with *Vernonia amygdalina*, *Carica papaya* and *Citrus x aurantifolia* while Eye ailment was for *Ocimum gratissimum* and *Telfairia occidentalis* is for boosting blood (Table 4). When the plant species were categorized into 29 families, it was observed that the most cited one was Asteraceae (10.26%), followed by the family Euphorbiaceae (7.69%) (Table 5). These two families have also been reported to be predominant in an ethnobotanical inventories carried out in some Southern parts of Nigeria (Uzodimma, 2013; Obata & Aigbokhan, 2012).

**Table 4:** Medicinal plants mentioned by the students and their uses

	Plant	Family	Common names	Main disease
1	*Acalypha wikesiana* Muell Arg	Euphorbiaceae	Acalypha	Skin infection
2	*Aframomum melegueta* K. Schum	Zingiberaceae	Alligator pepper	Wound
3	*Ageratum conyzoides* L.	Asteraceae	Goat weed	Eye
4	*Allium cepa* L.	Alliaceae	Onion	Fainting
5	*Aloe vera* (L.) Burm.f	Aloaceae	Aloe vera	Eye
6	*Ananas comosus Merr.*	Bromeliaceae	Pineapple	Measles
7	*Azdirachta indica* A. Juss	Meliaceae	Neem	Fever
8	*Bryophyllum pinnatum* (Lam) Oken	Crassulaceae	Never die	Cough
9	*Capsicum frutescens* L.	Solanaceae	Pepper	Wound
10	*Carica papaya* L.	Caricaceae	Pawpaw	Fever
11	*Chromolaena odorata* (L.) King & H.E Robins	Asteraceae	Christmas bush	Bleeding
12	*Citrus x aurantifolia* Burn.f.	Myrtaceae	Lime	Fever
13	*Citrus x sinensis* Osbeck	Rutaceae	Orange	Energy/appetite
14	*Cola nitida* (Vent.) Schott &Endl.	Sterculiaceae	Kola nut	Skin infection
15	*Corchorus olitorius* L.	Tiliaceae	Jute leaf	Skin beauty
16	*Costus afer* Ker Gawl	Costaceae	Monkey sugar cane	Chicken pox
17	*Cymbopogon citratus* DC Stapf.	Poaceae	Lemon grass	Fever
18	*Elaeis guineensis* Jacq.	Arecaceae	Palm kernel	Fever
19	*Ficus exasperata* L	Moraceae	Fig tree	Blood
20	*Garcinia kola* Heckel	Clusiaceae	Bitter cola	Cough
21	*Helianthus anuus* L	Asteraceae	sun flower	Bleeding
22	*Hibiscus esculentus* (L.) Moench	Malvaceae	Okro	Bite
23	*Ipomea batatas* L.	Convovulaceae	Potato	Pile
24	*Jatropha tanjorensis* Ellis &Saroja	Euphorbiaceae	Hospital too far	Blood
25	*Solanum lycopersicum* L	Solanaceae	Tomato	Blood
26	*Mangifera indica* L.	Anacardiaceae	Mango	Fever
27	*Manihot esculenta* Crantz	Euphorbiaceae	Cassava	Bite/inflammation
28	*Moringa oleifera* L.	Moringaceae	Moringa	Eye
29	*Musa paradisiaca* L.	Musaceae	Plantain	Chicken pox
30	*Ocimum gratissimum* L.	Lamiaceae	Scent leaf	Eye
31	*Pennisetum purpureum* L	Poaceae	Elephant grass	Fever
32	*Persea americana* Mill	Lauraceae	Avocadro pear	Arthritis
33	*Phyllanthus amarus* Schum. & Thonn.	Phyllathaceae	Phyllanthus	Labour induction
34	*Psidium guajava* L*.*	Rutaceae	Guava	Stomach ache
35	*Talinium trangulare* (Jacq.) Willd.	Portulacaceae	Water leaf	Pain
36	*Telfairia occidentalis* Hook. F	Cucurbitaceae	Fluted pumpkin	Blood
37	*Tetrapleura tetraptera* Taub.	Leguminosae-Mimosaceae	Tetrapleura	Ulcer
38	*Uvaria chamae* P. Beauv.	Annonaceae	Bush pepper	Skin infection
39	*Vernonia amygdalina* Delile	Asteraceae	Bitter leaf	Fever
	OTHERS			
40			Cray fish	Blood
41			Fresh egg	Blood
42			Honey	Arthritis
43			Male lizard	Cough
44			Snake	Skin rashes

**Table 5:** Medicinal Plant families mentioned by the students

Families	Occurrence	% Occurrence
Aliaceae	1	2.56
Aloaceae	1	2.56
Anarcadaceae	1	2.56
Annonaceae	1	2.56
Arecaceae	1	2.56
Asteraceae	4	10.26
Bromeliaceae	1	2.56
Caricaceae	1	2.56
Convovulaceae	1	2.56
Costaceae	1	2.56
Crassulaceae	1	2.56
Cucurbitaceae	1	2.56
Euphorbiaceae	3	7.69
Gutiferae	1	2.56
Lamiaceae	1	2.56
Lauraceae	1	2.56
Leguminosae-Mimosaceae	1	2.56
Malvaceae	1	2.56
Meliaceae	1	2.56
Moraceae	1	2.56
Moringaceae	1	2.56
Musaceae	1	2.56
Myrtaceae	1	2.56
Poaceae	2	5.13
Portulaccaceae	1	2.56
Rutaceae	2	5.13
Solanaceae	1	2.56
Sterculiaceae	1	2.56
Tiliaceae	1	2.56
Zingiberaceae	1	2.56
	39	

### 3.4 Disease Category

Fever was the most frequently mentioned singular disease with 43% citation having *Vernonia amygdalina* as the most cited plant species for it (37%). This agrees with the findings from Portharcourt metropolis in the Niger Delta Region, in which most of the plants cited were used to treat malaria fever, underlying the importance of this disease in the region (Nwazuoma & Dappa, 2013). Malaria has been reported as the world’s most important parasitic disease. Nearly half of her population is exposed to malaria. An estimate of 3.3 billion people was at risk in 2011 with Sub-saharan Africa having the highest risk (Kasali et al., 2014; Ibrahim et al., 2012; Adebayo & Kretti, 2011). It is endemic in Nigeria, accounting for a quarter of all cases in the Sub-saharan Africa (Omosun et al., 2013), with about 97% of the population at risk (FMOH, 2009). It also accounted for nearly 110 million clinically diagnosed cases of fever yearly and an estimate of annual infant and children under five mortality of about 25% and 30% respectively (FMOH, 2009). It is also responsible for an estimated maternal mortality of 11%. Apart from the direct health impact, the annual social and economic burden in form of treatment cost, prevention cost and loss of man hours, is estimated to be about 132 billion naira (Kunle et al., 2013). The Bayelsa State Ministry of Health has also identified malaria as the lead cause of 210 deaths recorded in the State in 2011. Out of the 210 cases of deaths that occurred in about 35 different diseases under the public health sector surveillance, malaria was said to have led the pack with 102 deaths in 2011 (Daily Trust, Feb. 28, 2012). Niger Delta Region experiences the highest amounts of rain fall in Nigeria receiving over 4000 mm (157.5 in) annually thus the terrain is characterized by marshy areas which encourage the breeding of mosquitoes. Next to fever in rank was eye ailment (9.1%), the most cited plant species for it was *Ocimum gratissimum* (10.8%). These were followed by Blood diseases (6.6%), Stomach ache (7%), Bleeding (4%) with the most cited plant species as *Telfairi aoccidentalis* (8.7%), *Citrus aurantifolia* (3.5%) and *Vernonia amygdalina* (6.9%) respectively (Table 6, Appendices 1a, b, and c). When the diseases were categorized, fever ranked highest among the categories of diseases cited by the respondents (45%), 13.5% of plant species were mentioned for this category. Next to it was haematological conditions which had 13% citation and 13.5% plant species mentioned for it. Others were gastrointestinal disorders (10.6%), ophthalmology (9.4%) and musculoskeletal ailments (6.4%) with 15 %, 6.8% and 13.5% number of species of plants cited respectively (Table 7).

**Table 6:** Category of ailments and most cited medicinal plants for treatment

s/n	Disease	Citation	% citation of disease	most cited plant (s)	citation of plant for Disease	% citation of plant for Disease
1	Acne	2	0.4	*Citrus aurantifolia*	2	0.9
2	Apetite	2	0.4	*Citrus sinensis*	1	0.4
				*Capsicum frutescens*	1	0.4
3	Arthritis	3	0.6	*Ocimum gratissimum*	1	0.4
				*Persea americana*	1	0.4
4	Asthma	1	0.2	*Allium cepa*	1	0.4
5	Bleeding	19	4	*Vernonia amygdalina*	16	6.9
6	Blood	31	6.6	*Telfairia occidentalis*	20	8.7
7	Body tonic	2	0.4	*Ocimum gratissimum*	1	0.4
8	Catarrh	6	1.3	*Ocimum gratissimum*	3	1.3
9	Chicken pox	3	0.6	*Musa paradisiaca*	1	0.4
				*Azadirachta indica*	1	0.4
				*Costus afer*	1	0.4
10	Conjunctivitis	1	0.2	*Musa paradisiaca*	1	0.4
11	Cough	11	2.3	*Garcinia kola*	2	0.9
12	Diabetes	4	0.9	*Vernonia amygdalina*	3	1.3
13	Diarrhoea	3	0.6	*Vernonia amygdalina*	1	0.4
				*Ocimum gratissimum*	1	0.4
14	Dislocation	1	0.2	*Capsicum frutescens*	1	0.4
				*Aframomum melegueta*	1	0.4
15	Dry throat	2	0.4	*Capsicum frutescens*	1	0.4
16	Ear infection	2	0.4	*Bryophyllum pinnatum*	1	0.4
17	Eczema	4	0.9	*Bryophyllum pinnatum*	4	1.7
18	Energy	5	1.1	*Telfairia occidentalis*	2	0.9
19	Eye	43	9.1	*Ocimum gratissimum*	25	10.8
20	Fainting	1	0.2	*Allium cepa*	2	0.9
21	Headache	9	1.9	*Ocimum gratissimum*	7	3
22	Hernia	1	0.2	*Tetrapleura tetraptera*	1	0.4
23	High Blood Pressure	3	0.6	*Moringa oleifera*	2	0.9
24	Inflammation	4	0.9	*Ocimum gratissimum*	1	0.4
				*Garcinia kola*	1	0.4
25	Labour induction	1	0.2	*Phyllanthus amarus*	2	0.9
26	Malaria	202	43	*Vernonia amygdalina*	85	36.8
27	Massaging	1	0.2	*Elaeisis guineensis*	1	0.4
28	Measles	6	1.3	*Vernonia amygdalina*	3	1.3
29	Menstrual pain	1	0.2	*Musa paradisiaca*	1	0.4
30	Navel healing	1	0.2	*Bryophyllum pinnatum*	1	0.4
31	Pain	11	2.3	*Citrus aurantifolia*	2	0.9
				*Moringa oleifera*	2	0.9
32	Pile	5	*1.1*	*Ipomea batatas*	4	1.7
33	Ringworm	1	0.2	*Aloe vera*	1	0.4
34	Skin beauty	2	0.4	*Telfairia occidentalis*	1	0.4
				*Cochorus olitorius*	1	0.4
35	Skin infection	11	2.3	*Ocimum gratissimum*	5	2.2
36	Snake/ scorpion Bite	4	0.9	*Manihot esculenta*	2	0.9
				*Hibiscus esculentus*	2	0.9
37	Stomach ache	33	7	*Citrus aurantifolia*	8	3.5
38	Stomach ulcer	6	1.3	*Tetrapleura tetraptera*	3	1.3
39	Typhoid	11	2.3	*Carica papaya*	6	2.6
40	Weight control	3	0.6	*Ocimum gratissimum*	1	0.4
				*Cochorus olitoriu*	1	0.4
				*Citrus aurantifolia*	1	0.4
41	Worm expeller	1	0.2	*Citrus aurantifolia*	1	0.4
42	Wound	6	1.3	*Ocimum gratissimum*	4	1.7

**Table 7:** Categories of Prevalent diseases in the study area

	Disease category	citation	% citation	Number of specie	% Number of specie
1	Cardiovascular diseases	3	0.6	2	1.5
2	Dermatology	33	7	20	15
3	Fever	213	45.3	18	13.5
4	Gastrointestinal tract	50	10.6	20	15
5	Haematology	61	13	18	13.5
6	Metabolic diseases	12	2.6	9	6.8
7	Musculoskeletal	30	6.4	18	13.5
8	Obstetrics and Gynaecology	3	0.6	2	1.5
9	Ophthalmology	44	9.4	9	6.8
10	Respiratory /Ear Nose & throat	22	4.7	15	11.3
11	Structural diseases	2	0.4	2	1.5
		473		133	

### 3.5 Plant’s Habits and Plant Parts Used As Medicines

**Figure 2:**
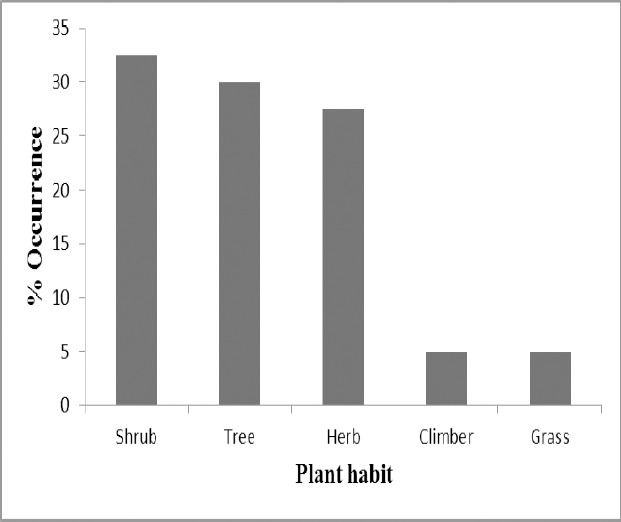
Different plant habits of the medicinal plants mentioned by the students

**Figure 3:**
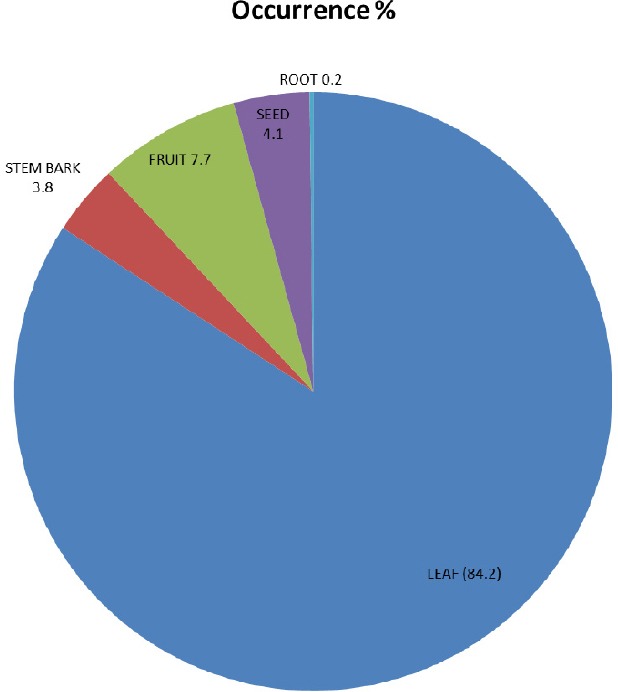
The plant parts used for their medicinal value mentioned by the students

Approximately 33% of the herbal medicines mentioned were from shrub, 30% were obtained from trees, 27.5% from herbs while 10% were shared equally between grass and climbers (Table 8 and Figure 2). Majority (84%) of the herbal medicines mentioned were obtained from leaf while root produced the least (0.2%) (Figure 3). The use of leaves could be justified by the abundance of chemical groups they contain. In fact, leaves are known as the main synthesis site of secondary metabolites in plants and are the most commonly used plant parts by traditional medicine practitioners (Katemo et al., 2012; Lavergne & Vera, 1998; Idowu et al., 2010; Pousset, 1989; Moswa, 2005). This also constitutes an advantage as harvesting leaves on a sustainable manner ensures continuity of the plant.

**Table 8:** Plant habits

Medicinal palnts	Family	Habits
*Acalypha wikesiana* Muell Arg	Euphorbiaceae	Shrub
*Aframomum melegueta* K. Schum	Zingiberaceae	Herb
*Ageratum conyzoides* L.	Asteraceae	Shrub
*Allium cepa* L.	Aliaceae	Herb
*Aloe vera* (L.) Burm.f	Aloaceae	Shrub
*Ananas comosus Merr.*	Bromeliaceae	Herb
*Azdirachta indica* A. Juss	Meliaceae	Shrub
*Bryophyllum pinnatum* (Lam) Oken	Crassulaceae	Herb
*Capsicum frutescens* L.	Solanaceae	Shrub
*carica papaya* L.	Caricaceae	Tree
*Chromolaena odorata* (L.) King & H.E Robins	Asteraceae	Shrub
*Citrus aurantifolia* Burn.f	Myrtaceae	Tree
*Citrus sinensis* Osbeck	Rutaceae	Tree
*Cola nitida* (Vent.) Schott &Endl.	Sterculiaceae	Tree
*Corchorus olitorius* L.	Tiliaceae	Herb
*Costus afer* Ker Gawl	Costaceae	Shrub
*Cymbopogon citratus* DC Stapf.	Poaceae	Herb/Grass
*Elaeis guineensis* Jacq.	Arecaceae	Tree
*Ficus exasperata* L	Moraceae	Tree
*Garcinia kola* Heckel	Gutiferae	Tree
*Hibiscus esculentus* (L.) Moench	Malvaceae	Herb
*Ipomea batatas* L.	Convovulaceae	Climber
*Jatropha tanjorensis* Ellis & Saroja	Euphorbiaceae	Shrub
*Solanum lycopersicum* L.		Herb
*Mangifera indica* L.	Anarcadiaceae	Tree
*Manihot esculenta* Crantz	Euphorbiaceae	Shrub
*Moringa oleifera* L.	Moringaceae	Tree
*Musa paradisiaca* L.	Musaceae	Shrub
*Ocimum gratissimum* L.	Lamiaceae	Shrub
*Pennisetum purpureum* L.	Poaceae	Herb/Grass
*Persea americana* Mill	Lauraceae	Tree
*Phyllanthus amarus* Schum. & Thonn.	Euphorbiaceae	Herb
*Psidium guajava* L*.*	Rutaceae	Tree
*Talinium trangulare* (Jacq.) Willd.	Portulaccaceae	Herb
*Telfairia occidentalis* Hook. F	Cucurbitaceae	Climber
*Tetrapleura tetraptera* Taub.	Leguminosae-Mimosaceae	Tree
*Uvaria chamae* P. Beauv.	Annonaceae	Shrub
*Vernonia amygdalina* Delile	Asteraceae	Shrub

### 3.6 Similarity of Use

Plants like *Vernonia amygdalina*, *Ocimum gratissimum*, *Carica papaya*, *Mangifera indica*, *Citrus x aurantifolia* and *Azadirachta indica* which were mentioned in this study for fever have also been reported in the literature for use as such (Ezekwesili, 2004; Ige, 2011; Challand & Willcox; 2009). However, the use of *Vernonia amygdalina* which was the most cited for fever is not very common in most studies in Nigeria (Dike et al., 2012; Obata & Aigbokhan, 2012). Plants like *Mangifera indica* and *Azadirachta indica* are not commonly available in the Niger Delta as in the other parts of Nigeria; this may be the reason for their being less mentioned. Most of the leaves of *Azadirachta indica* being used for the treatment of malaria are obtained from the markets. Majority of these students have not seen the cultivated tree before.

### 3.7 Miscellaneous Sources of Medicines

A few respondents in addition to various plant species mentioned, cited oil from snakes, raw cray fish, raw eggs and male lizards as sources of medicines. Traditional medicines are diverse health practices, approaches, knowledge and beliefs that incorporate animal in addition to plants (Oreagba & Oshikoya, 2011).

### 3.8 Mode of Preparation of Recipes

Water is the most used solvent for the preparation of the recipes (Table 9). This confirms the reports of several other authors (Kasali, 2014; Dibong et al., 2011; Saoud et al., 2010). In fact, water is the cheapest and the most available solvent that can dissolve a high number of metabolites and high temperature permits a rapid extraction of active ingredients. However, some of these metabolites can be degraded by heat. Salts were added occasionally probably as a preservative especially when the solvent of preparation was water. Illicit gin was also sometimes used and in this case salt would be unnecessary.

**Table 9:** Method of preparation of Plants

	Plant	Uses	Method of preparation	Mode of application/Dosage
1	Bitter leaf	Fever	Crushed in water/chew leaf	Internal use; 1 shot x 3 till recovery
		Wound	Mix juice with soap	External; x 2 till recovery
		Eye	Crushed and apply juice	External; 1 drop x 2 till recovery
2	Pawpaw	Typhoid fever/malaria	Decoction	Internal; 1 shotx 1 for a week/
				Internal; Inhale at night
3	Mango	Malaria fever	Decoction, salt may be added	Internal;1 shot x 2 for 1week
4	Scent leaf	Eye	Crushed leaf and apply juice	External; apply as needed
		Wound	Crushed and apply juice, salt/palm oil may be added	
		Oral use	Concoction	Internal use; 1 shot x 3 till recovery.
		Catarrh	Decoction	Inhalation; at night as needed
5	Never die	Eye	Crushed leaf and apply juice	Eye; 2 drops x 3
		Stomach ache	Chew	Chew one or two leaves as needed
6	Lemon grass	Fever	Decoction	1shot twice for 1 week
7	Moringa leaf	Pain	Infusion	1-2 teaspoonful thrice daily,
				1-2 seeds thrice daily
8	Fluted pumpkin	Blood	Chew leaf, grind seed in water and infuse, beverage can be added	1 glass as needed
9	Lime	Malaria	Extract juice in water/ add pawpaw leaf, mango stem bark/leaves and boil	External; Bathe as required inhale, I up twice daily orally
		Weight loss	mix juice with honey	Internal; One glass a day until result shows
		Malaria	Infuse leaf/fruit / lick	Internal; One glass a day until result shows
10	Alligator pepper	Cough	Chew seed	1-2 seedsas needed
11	Aloe vera	Juice/skin	Add salt to bitter leaf and extract juice and mix with juice	External; Apply twice daily
		Oral	Boil leaf	Internal; 1 tea cup with corn pap twice daily
		Eye	Squeeze juice	Instil into the eyeonce a day
		Labour induction	Chew	Internal; Chew leaf once a day for two weeks
12	Kola nut	Skin infection	Decoction of root	External; Bathe with it twice daily
13	Cassava	Pain	Chew leaf with gin	Internal; Chew twice daily
			Pound fresh leaf with ash	External; Rub twice daily
14	Plantain	Chicken pox/measles	Collect sap	External; Apply twice daily on skin for measles/ 1 shot once daily orally
15	Guava	Cough/ulcer	Chew aerial part	Internal; chew 3-4 leaves as needed
16	Tetraptera	Ulcer	Mix with alligator pepper /Tincture	Internal; 1 shot daily until relief
17	Orange	Energy/appetite	Add leaf to lime / Decoction	Internal; 1 shot thrice daily
18	Jute leaf	Skin beauty	Decoction	Internal; Drink like tea
19	Fig tree	Blood	Soak in water	Internal; Drink like tea
20	Acalypha	Skin infection	Decoction	Internal; 1 spoonful twice daily/
				External; bathe with it
21	Neem	Malaria	Add scent leaf, bitter leaf and lime/Tincture	Internal; 1 shot thrice daily until relief
22	Christmas bush	Bleeding	Squeeze leaf	External; Apply juice on affected part
23	Palm kernel	Fever	Extract oil from seed	External; Apply/rub at night
24	Avocadro pear	Arthritis	Infusion/honey may be added	External; 1 glass once a day until result shows
25	Pepper	Fever/wound	Decoction/pound the seed	Internal; 1 shot twice daily/
				External: apply pounded seed twice daily
26	Sunflower	Bleeding	Squeeze juice	External; Apply as needed
27	Never die	Cough	Heat the leaf and extract juice	Internal; 1 teaspoonful juice twice a day
				External; apply to the skin twice a day/ massage
28	Male lizard	Cough	Concoction	Internal; Take once
29	Snake	Skin rashes	Extract oil	External; Rub twice daily
30	Elephant grass	Fever	Decoction	Internal; 1 shot twice daily
31	Potato	Measles	Decoction	Internal; 1 spoonful thrice daily
32	Bush pepper	Skin infection	Chew seed	Internal; Chew 1-2 seeds daily
33	Bitter cola	Cough	Chew and drink illicit gin	Internal; 1 – 2 seed twice daily
34	Tomato	Blood	Add to ugu juice	Internal; 1 cup as required
35	Onion	Fainting	Extract juice/ extract leaf with beverage	Eye; Apply to the eye
				Internal; 1 cup as needed in asthma
36	Water leaf	Blood	Extract in water	Internal; 1 shot thrice daily
37	Cray fish	Blood	Fresh	Eat fresh once daily
38	Goat weed	Eye	Squeeze	Eye; Apply to the eye once daily
39	Okro	Bite/sting	Squeeze	External; Apply to the affected area
40	Pineapple	Measles	Boil fruit peels	External: Bathe with it until relief
41	Monkey sugar cane	Chicken pox	Extract juice	External; Apply to the skin
				Internal; 1 teaspoonful twice daily
42	Fresh egg	Blood	Mix with beverage	Internal; 1 cup as needed
43	Hospital too far	Blood	Extract in water	Internal; 2 glasses daily
44	Phyllanthus	Labour induction	Extract in water	Internal; 1 cup as required

## 4. Conclusion

There is the need to preserve the indigenous knowledge of herbal medicines; this can be done by inclusion of herbal medicine study in school’s curriculum. This inclusion will impart the indigenous knowledge in the pupils as they have an advantage of preserving the indigenous knowledge by carrying on the practice of traditional medicine to their old age. Through formal training, herbal medicine will be accorded more recognition and taken more seriously. This will increase awareness in the use of simple, harmless but useful herbs. It will also encourage some of the pupils to study herbal medicine or related courses to promote herbal medicine in the country. Now that Nigeria is preparing to legalize herbal medicine to her conventional healthcare system, studying herbal medicine from SS 2 will strengthen the system of medicine which may be incorporated into the healthcare system of the country.

## Appendix

**Appendix 1a:** Cited plants and the diseases for which they are used

Plant	Cough	body	energy	pain	worm	acne	skin	ulcer	hernia	apetite	dislo	dry	labour	chicken	massaging

		tonic			Expeller		beauty				Cation	throat	induction	pox	
*Vernonia amygdalina*															
*Ocimum gratissimum*	1	1													
*Carica papaya*	1		1												
*Mangifera indica*			1												
*Cymbopogon citratus*				1											
*Pennisetum purpureum*															
*Citrus x aurantifolia*				2	1	2									
*Psidium guajava*	1		2												
*Telfairia occidentalis*							1								
*Aframomum melegueta*	1							2							
*Tetrapleura tetraptera*								3	1						
*Uvaria chamae*		1		1						1	1	1			
*Helianthus anuus*															
*Talinium trangulare*				2											
*Uvaria chamae*												1			
*Acalypha wikesiana*															
*Aloe vera*								1					1		
*Azadirachta indica*														1	
*Chromolaena odorata*															
*Elaeis guineensis*															1
*Musa paradisiaca*														1	
*Citrus x sinensis*			1							1					
*Corchorus olitorius*							1								
*Ficus exasperate*															
*Allium cepa*															
*Manihot esculenta*				1											
*Hibiscus esculentus*															
*Ananas comosus*															
*Costus afer*														1	
*Garcinia kola*	2														
*Moringa oleifera*				2											
*Bryophyllum pinnatum*	1			2											
*Ipomea batatas*															
*Ageratum conyzoides*															
*Solanum lycopersicum*															
*Persea Americana*															
Honey															
male lizard	4														
cray fish															
Snake															
*Cola nitida*															
fresh egg															
*Jatropha tanjorensis*															
*phyllanthus amarus*													2		
	11	2	5	11	1	2	2	6	1	2	1	2	3	3	1

**Appendix 1b:** Cited plants and the diseases for which they are used

Plant	menstrual	conjunc	asthma	fainting	bite	BP	eczema	ear	navel	skin	ring
	pain	tivitis							healing	rashes	worm
*Vernonia amygdalina*											
*Ocimum gratissimum*											
*Carica papaya*											
*Mangifera indica*											
*Cymbopogon citratus*											
*Pennisetum purpureum*											
*Citrus x aurantifolia*								1			
*Psidium guajava*											
*Telfairia occidentalis*											
*Aframomum melegueta*											
*Tetrapleura tetraptera*											
*Uvaria chamae*											
*Helianthus anuus*											
*Talinium trangulare*											
*Uvaria chamae*											
*Acalypha wikesiana*											
*Aloe vera*						1					1
*Azadirachta indica*											
*Chromolaena odorata*											
*Corchorus olitorius*											
*Ficus exasperate*	1	1									
*Allium cepa*											
*Manihot esculenta*											
*Ficus exasperate*											
*Allium cepa*			1	2							
*Manihot esculenta*					2						
*Hibiscus esculentus*					2						
*Ananas comosus*											
*Costus afer*											
*Garcinia kola*											
*Moringa oleifera*						2					
*Bryophyllum pinnatum*							4	1	1		
*Ipomea batatas*											
*Ageratum conyzoides*											
*Solanum lycopersicum*											
*Persea Americana*											
Honey											
male lizard											
cray fish											
Snake										1	
*Cola nitida*											
fresh egg											
*Jatropha tanjorensis*											
*phyllanthus amarus*											
	1	1	1	2	4	3	4	2	1	1	1

**Appendix 1c:** Cited plants and the diseases for which they are used

Plant	fever	cattarh	blood	stomach	bleed	inflam	measles	eye	head	diarr	diab	clean	skin	weight	arthr	wound	typhoid	pile
					ache	ing	mation			ache	hoea	etes	ser	infection	control	itis			
*Vernonia amygdalina*	85	3	1	4	16	1	3	7	2	1	3	2						
*Ocimum gratissimum*	17	2		7				25	7	1	1		5	1	1	4	1	1
*Carica papaya*	31			2									1				6	
*Mangifera indica*	15			1														
*Cymbopogon citratus*	18	1		1														
*Pennisetum purpureum*	2																	
*Citrus x aurantifolia*	16			11			1							1			1	
*Psidium guajava*	1			4														
*Telfairia occidentalis*	1		25															
*Aframomum melegueta*	-							1					1					
*Tetrapleura tetraptera*	-																	
*Uvaria chamae*	1															2		
*Helianthus anuus*					2													
*Talinium trangulare*			1															
*Uvaria chamae*													1					
*Acalypha wikesiana*																		
*Aloe vera*	1							3					2					
*Azadirachta indica*	12																3	
*Chromolaena odorata*					1													
*Corchorus olitorius*	1																	
*Ficus exasperate*																		
*Allium cepa*	1																	
*Manihot esculenta*														1				
*Ficus exasperate*			1															
*Allium cepa*								1										
*Manihot esculenta*				1		2												
*Hibiscus esculentus*																		
*Ananas comosus*							1											
*Costus afer*																		
*Garcinia kola*						1												
*Moringa oleifera*				2				2				2						
*Bryophyllum pinnatum*								3										
*Ipomea batatas*							1											4
*Ageratum conyzoides*								1										
*Solanum lycopersicum*			1															
*Persea Americana*															1			
Honey															1			
male lizard																		
cray fish			1															
Snake																		
*Cola nitida*										1			1					
fresh egg			1															
*Jatropha tanjorensis*			1															
*phyllanthus amarus*																		
	202	6	32	33	19	4	6	43	9	3	4	4	11	3	3	6	11	5
